# Impact of COVID-19 Restrictions in Childbirth and Puerperium: A Cross-Sectional Study

**DOI:** 10.3390/healthcare11020249

**Published:** 2023-01-13

**Authors:** María Suárez-Cortés, María de los Ángeles Castaño-Molina, Antonio Jesús Ramos-Morcillo, Alonso Molina-Rodriguez, Ismael Jiménez-Ruiz, María Jesús Hernández-López, Francisco David Harillo-Acevedo, César Carrillo-García

**Affiliations:** 1 Servicio Murciano de Salud, 30120 Murcia, Spain; 2 Faculty of Nursing, University of Murcia, 30120 Murcia, Spain

**Keywords:** COVID-19, parturition, maternal–fetal relations

## Abstract

Introduction (1): The COVID-19 pandemic led to changes in healthcare during pregnancy, childbirth and puerperium. The objective of this study was to know the impact of visit restrictions, PCR performance and use of masks on delivery and puerperium care. Methods (2): A descriptive cross-sectional study was carried out. A survey was used to assess the impact of COVID-19-related measures on women who had given birth in hospitals in the Region of Murcia, Spain, between March 2020 and February 2022. Results (3): The final sample size was 434 women. The average scores were 4.27 for dimension 1 (Visit restrictions), 4.15 for dimension 2 (PCR testing) and 3.98 for dimension 3 (Mask use). More specifically, we found that the restriction of visits was considered a positive measure for the establishment of the mother–newborn bond (mean score 4.37) and that the use of masks at the time of delivery should have been made more flexible (mean score 4.7). Conclusions (4): The policy of restricting hospital visits during the pandemic caused by COVID-19 has been considered beneficial by mothers, who expressed that they did not feel lonely during their hospital stay.

## 1. Introduction

The process of childbirth and puerperium and everything that happens around it directly influence the process of becoming a mother, and the mother–child bond. In addition, the birth of a child causes changes in the dynamics of the couple and in the relationship of the whole family [1,2,3].

During the pandemic caused by COVID-19, healthcare during pregnancy, childbirth and puerperium has undergone a series of adaptations according to the level of risk at each moment. These adaptations of the protocols directly influence the care provided to women [4].

Key measures taken during the COVID-19 pandemic in relation to delivery care include (a) restriction of visiting hours, (b) mandatory facemask use and (c) diagnostic testing for active infection [5].

These measures and restrictions, together with the fear of health professionals, have distorted the perception of women in relation to their pregnancy, childbirth and puerperium process [6,7]. However, throughout the pandemic, the increase in knowledge of the disease, its transmission mechanisms, evolution of treatment and vaccination has helped to add weight the results and change health actions, as well as the rules of hospital access [8,9,10].

The consequences of restrictions on family visits have been diverse, affecting both the mother’s discharge from hospital and the delay in the other family members getting to know the baby. However, the restriction of visits may have been seen as something positive by some women, because a calm and relaxed environment contributes to the correct establishment of maternal bonding and a higher rate of breastfeeding [11,12].

There is extensive literature on the benefits of accompaniment throughout the process of childbirth and puerperium [13,14,15]. As examples at the national level in Spain, we have the *Guide to Normal Childbirth Care* published by the Ministry of Health in 2010 [16] and promoted by the Strategy for Normal Childbirth in 2008 [17], as well as the document “El Plan de Parto y Nacimiento” developed by the Ministry of Health in 2011 [18]. In the international field, we can find the recommendations contained in the “WHO recommendations for care during childbirth, for a positive birth experience” [19] and the recommendations contained in “Promoting Effective Perinatal Care 2002 Essential Newborn Care and Breastfeeding Training modules WHO Regional Office for Europe” [20], all of which suggest that it is in the best interest of the mother and the newborn to allow the person of the mother’s choice to accompany her at the time of birth. In the Region of Murcia, Spain, this recommendation has been followed since. Although visits were restricted, a companion was allowed at all times, as established by national and international recommendations [16,17,18,21,22,23].

In contrast, the effect of visits in the hospital environment has not been investigated, so we consider it necessary to study how recent the restriction of visits has affected women. 

The aims of this study were to determine the impact of COVID-19 measures in relation to childbirth and puerperium care in mothers attended to in hospitals in the Region of Murcia, Spain. The specific objective of the study was to determine the impact on delivery and postpartum care of the restriction of visits, the use of the mask and the use of diagnostic tests for active infection on admission.

The Region of Murcia is in the southeast of Spain, and it has a total of ten public hospitals. Deliveries are attended in six of these hospitals, where midwives were the professionals who attended the normal deliveries of both COVID-19 positive and negative women, following the clinical practice guideline for normal delivery care [16].

## 2. Materials and Methods

### 2.1. Study Design

Descriptive cross-sectional study on the impact of the different measures implemented during the pandemic caused by COVID-19 in the Region of Murcia, Spain, on the process of childbirth and puerperium.

### 2.2. Participants

Non-probabilistic snowball sampling was used. The questionnaire was disseminated online through WhatsApp and Telegram groups of women and through the social networks (Instagram and Facebook) of several midwives with professional profiles. Among those that stand out, the Instagram profile @tusmatronascontigo, which was created during the COVID-19 pandemic by all primary care midwives in the Region of Murcia, and the profile @matronasmurcia, which belongs to the association of midwives in the Region of Murcia [24].

Participants were women who gave birth in the Region of Murcia between 1 March 2020 and 28 February 2022. 

Inclusion criteria were (1) to be of legal age, (2) to understand Spanish and (3) to have given birth in a hospital in the Region of Murcia.

### 2.3. Instruments

Data sampling methods were as follows:

A questionnaire of sociodemographic and clinical variables, which included date of delivery, nationality, maternal age, level of education, parity, weeks of gestation, induction of labor, type of delivery and having suffered COVID-19 infection, was utilized. 

To assess the impact of the COVID-19 measures, an “ad hoc” survey was developed, with 3 dimensions and a total of 10 items with a 5-point Likert scale (1 indicating strong disagreement and 5 strong agreement). Dimension 1, “Visit restrictions”, measures the impact of not having visits during the postpartum period in hospital. It is composed of items 1, 2 and 3, with a maximum score of 15 and a minimum score of 3. Dimension 2, “PCR testing”, measures women’s opinions about the performing of PCR tests in the hospital environment and is composed of items 6, 7 and 8, with a maximum score of 15 and a minimum score of 3. In addition, 2 more items were added: item 4, “I found myself alone during my hospital stay, missing my loved ones,” and item 5, “I consider that the postpartum period has been more relaxed than in previous deliveries due to the visit restrictions.” This last item was to be answered exclusively by women who had more children.

A first version of the survey was created by 3 experts in health sciences research who had more than 10 years of experience. The items were selected to include the main changes that occurred in the care of women during childbirth during the COVID-19 pandemic. This initial survey was presented to the postpartum unit supervisor and the breastfeeding support midwife, who suggested additional items (4 and 5 in the final survey) to widen the scope of the questions regarding the psychological well-being of the mother during their stay at the hospital.

To obtain content validity, 10 midwives were used, resulting in an adequate content validity index for individual items (item-wise CVI between 0.8 and 1), as well as for the whole questionnaire (scale-wise CVI = 9) (Table S1). Regarding the reliability analysis, the internal consistency (Cronbach’s α) of the scale based on the scores of the participants in the survey was analyzed, with a value of α = 0.842 obtained for dimension 1, a value of α = 0.715 for dimension 2, a value of α = 0.683 for dimension 3 and, for the total scale, a value of α = 0. 748 [25].

### 2.4. Data Collection

A data collection form with the measurement instruments described in the previous section was created using Google Forms. This form was disseminated by midwives with influence on the social networks Instagram and Facebook, as well as WhatsApp and Telegram messaging services. 

Data collection took place between 1 April and 7 April 2022.

### 2.5. Data Analysis

Data analysis was carried out with the statistical package JAMOVI version 2.3.9 for Windows. Statistical significance was established at *p* < 0.05. In the descriptive analysis of the sociodemographic questionnaire, frequencies and percentages were calculated for each of the categorical variables, whereas for quantitative variables, mean and standard deviation were calculated. To analyze the differences in the scores of the items and dimensions of the questionnaire between the women who had had COVID-19 and those who had not, Student’s t test for independent samples was used. On the other hand, one-factor ANOVA was used to analyze the differences in scores of the items and dimensions of the questionnaire according to the variables of level of education and type of delivery. The assumptions of normality and homoscedasticity were met. Finally, a post-hoc analysis was performed using Tukey’s test. Before applying the tests, its normality was checked with the Kolmogorov–Smirnov test, and its homoscedasticity was checked with Levene’s test.

### 2.6. Ethical Considerations

Approval for this study was obtained from the Ethics and Research Committee of the Hospital Virgen de la Arrixaca (code: 2022-2-7-HCUV; approved 29 March 2022). Data confidentiality and anonymity were guaranteed in agreement with current legislation on personal data protection. All the ethical principles of the Declaration of Helsinki were followed in this study [26].

## 3. Results

The final sample was 434 women, of the approximately 27,500 women who gave birth during the study period, of whom 47.3% had given birth at the Hospital Clínico Universitario Virgen de la Arrixaca, followed by 28.9% at the Hospital General Universitario Santa Lucia. Baseline characteristics of the studied sample are shown in Table 1.

The descriptive statistics analysis of the survey items is shown in Table 2. We can observe that for dimension 1, “Hospital visits”, the three items obtain a score of more than 4 out of 5. The average score for this dimension was 4.27.

In the additional question on whether they had felt lonely during their hospital stay, we can see that the mean score was 2.76, which indicated that they did not feel lonely.

In the question asked only to women who had already had another pregnancy on whether they considered that the postpartum period was more relaxed than in previous births because of the limitations of the visits, a score of 4.18 out of 5 was obtained, indicating a high agreement that their postpartum period had been more relaxed than in previous births.

On the other hand, regarding dimension 2, “PCR testing”, we observed that the four items obtained a score of 4 out of 5, which indicated that the mothers agreed with the performance of PCR tests on admission to hospital, and that this gave them peace of mind.

Finally, in dimension 3, “Use of mask”, we observed that the scores were lower compared to the other two dimensions, as it obtained a mean score of 3.94 (SD = 0.669), although the score was medium-high. However, in this dimension, the item with the highest score was item 12 (M = 4.70, SD = 0.67), where the women expressed their agreement with the flexibility on the use of the mask during labor.

When comparing women who had passed COVID-19 infection with those who had not, statistically significant differences were found in five items. In items 1, 5, 8 and 11, women who had not passed COVID-19 infection scored higher than those who had passed it, showing more agreement with restricting visits (*p* = 0.002) and PCR testing before hospital admission (*p* = 0.004), greater peace of mind to know that their PCR was negative (*p* = 0.011), and stronger belief that the use of a mask in the room was necessary when the rooms were shared with other pregnant women (*p* = 0.016). However, in item 9, women who had passed the COVID scored higher than those who had not, considering that a mask should be used in the case of not having a PCR or being positive in COVID-19 (*p* = 0.047) (Table 3).

When the results were compared according to the type of delivery, statistically significant differences were only obtained in item 4, “I was lonely during my hospital stay, missing my loved ones” (*p* = 0.048). The post-hoc analysis showed that the women who had had a caesarean section scored higher compared to women who had had a eutocic delivery (*p* = 0.035) (Table 4).

Finally, we analyzed the differences according to the variable level of studies. Statistically significant differences were found in item 4, “I felt lonely during my hospital stay, missing my loved ones” (*p* = 0.031), and item 8, “Knowing that my PCR was negative gave me peace of mind” (*p* = 0.001). The post-hoc analysis showed that in item 4, women with basic studies felt more loneliness than women with university studies (*p* = 0.021). In item 8, it was observed that women with university education scored lower compared to women with basic education (*p* = 0.046).

## 4. Discussion

The results of our study show that women understand the measures imposed by the COVID-19 pandemic situation and even consider that not having had visits during the puerperium was positive for their delivery process and for improving the mother–baby bond, because women scored item 3 with 4.37 points out of 5. However, regarding the use of masks, most women considered that their use should have been made more flexible at the time of delivery. These results are congruent with other studies that emphasize the importance of mother–child bonding for the establishment of breastfeeding and postpartum recovery [2,3,11,12]. 

Regarding the restriction on visits, the results of the study showed that most women (86.4%) agreed with the measure. They even considered this measure as positive for the birthing process (80.2%) and the establishment of the mother–newborn bond (86.4%). On the other hand, 36.2% of the women stated that they felt lonely during their stay at the hospital, although we cannot determine to what extent this was due to COVID-associated restriction on visits. Interestingly, it was found that women with higher education felt less lonely than women with basic education, and that women who gave birth by cesarean section felt lonelier during their hospital stay. This may be due to the fact that after surgery, women need more help and support from family members [27], in addition to the fact that it is a risk factor for a decrease in the rate of exclusive breastfeeding at discharge [28]. 

We have not found studies exploring the effect of an absence of visits during hospital stay. The published studies regarding visitation restrictions during hospital stay for childbirth during the COVID-19 pandemic explore the lack of continuous support by a person of the woman’s choice during the hospital delivery and puerperium process, as well as the lack of home visitation [29,30,31,32,33]. However, we found other studies that relate the lack of visits during the hospital stay with an improvement in establishment of breastfeeding and development of the mother–newborn bond [6,7]. During the initial stages of the COVID-19 pandemic, the Spanish Government published the document “Information and general guidelines for pregnant women in confinement” [4], which specifies that the time in the hospital after delivery is a good time to stimulate the establishment and development of the bond with your baby and to initiate breastfeeding because visits are restricted. In this same guide, in the recommendations to avoid COVID-19 infection at home, the importance of the process of becoming a mother is mentioned again. 

It is important to highlight that in our study, as in other similar studies [13,14], women considered that they have benefited from not having visits during the postpartum period in the hospital due to the restrictions imposed by the pandemic, because it allowed them to find a space without external interference to enjoy the tranquility with their companion and child, increasing their rest and recovery, enhancing breastfeeding and favoring bonding while making them feel more protected against COVID-19 [13,14,34].

In this regard, it should be noted that in recent years, maternity units have allowed unrestricted visits, possibly disregarding maternal well-being and increasing the risk of infection for the newborn. Perhaps it is time to consider models of controlled visits, such as those that have been implemented in several neonatal units in Spain [35,36,37], thus contributing to increased maternal rest, breastfeeding and development of bonding. 

Regarding the use of PCR tests, most women (82.3%) considered that the performance of a PCR on admission to hospital was a good measure. This was one of the first measures adopted during the pandemic and is still in place today [38]. In the hospitals of the Region of Murcia (Spain), a PCR is performed on every person admitted, but it was not mandatory for the accompanying person. In our study, we observed that 80.4% of the women believed that PCR should have been performed on those accompanying them. This desire on the part of the women to also know whether their companions were infected could be related to the feeling of security they felt knowing that the PCR they had been given was negative. 

Finally, with respect to the use of the mask, no evidence has been found on the perception of the use of the mask during delivery or postpartum by mothers. The studies carried out focus on the use of masks by professionals as preventive measures [39]. The use of masks in hospitals has evolved during the pandemic according to the available evidence [40,41]. Legislation on the use of masks in Spain is currently regulated by Royal Decree 115/2022 of 8 February [42], which eliminated the obligation to wear masks outdoors except at mass events in which the safety distance cannot be maintained. Subsequently, Royal Decree 286/2022, of April 19 [43], modified this rule for indoor spaces, leaving the decision on the use of masks in the work environment to the prevention committees. However, the use of masks in healthcare centers is still mandatory. Thus, currently in Spain, women must wear masks at the time of delivery and throughout their hospital stay. However, most of the women in the study (94.7%) stated that the use of the mask should be made more flexible at the time of delivery. This relaxation in the use of the mask during labor would coincide with the recommendations of FIGO (International Federation of Gynecology and Obstetrics, Spain) [44].

On the other hand, 67.3% of the women answered that they “agreed” or “strongly agreed” that the obligatory use of the mask should be maintained in case of unknown PCR status or active infection. Moreover, 60.1% of the women perceived the use of mask as a wise measure due to the pandemic situation, and the majority (67.3%) considered that its use was necessary in shared-use rooms. 

### Limitations

There are some limitations in this study. First, only women on the social networks were able to participate; this may have contributed to our sample being biased in reference to socioeconomic groups. In addition, we do not have data on the reasons for inductions or caesarean sections, something that can affect biopsychosocial well-being. In the present study, no assessment of test–retest or temporal stability and construct validity was conducted, so it was not possible to verify this aspect of reliability and validity. Finally, we should be aware that the sample studied cannot be representative of the reference population, and this can evidently affect the generalization of the results.

## 5. Conclusions

The policy of restricting hospital visits during the pandemic caused by COVID-19 has been considered beneficial for mothers, who expressed not feeling lonely during their hospital stay. In addition, it was found that women with higher education felt less lonely than women with basic education, and that women who gave birth by cesarean section felt lonelier.

The performance of PCR on admission to hospital was a measure widely accepted by the women, and they would even like to have it performed on their companions also.

The use of masks in the hospital environment is considered a good measure by the women, even more so when the rooms are shared. However, it would be advisable to review health policies to make its use more flexible during childbirth.

We consider that the most important finding of our research is the good acceptance by women of the restriction of visits, which should lead to relevant clinical implications.

## Figures and Tables

**Table 1:** Baseline characteristics of studied sample.

	*n* = 434	Frequencies	Percentage
Nationality	Spanish	421	97.5%
	Non-Spanish	13	2.5%
Level of Studies	University	290	67.6%
	Vocational training	104	24.2%
	Basic Studies	35	8.2%
Weeks of gestation		M = 39.3	SD = 1.52
Parity	Primiparous	268	61.9%
	multiparous	165	38.1%
Mode of delivery	Normal Birth	247	56.9%
	Instrumental delivery	96	22.1%
	Caesarean	91	21%
Induced delivery	Yes	166	22.1%
	No	267	77.9%
COVID-19	Positive	176	40.6%
	Negative	258	59.4%
Weeks of gestation is reported as mean (M) and standard deviation (SD)			

**Table 2:** Descriptive statistics for the survey items and dimensions.

					*n* (%)				
Items	N	Min. Max.	M (SD)	Med.	1	2	3	4	5
Dimension 1. Visit restrictions.	434	1 5	4.27 (0.910)	4.67					
1. I believe that the measure to limit visits is necessary because of the COVID-19 pandemic.	434	1 5	4.29 (0.994)	5	8 (1.8%)	35 (8.1%)	16 (3.7%)	137 (31.6%)	238 (54.8%)
2. I feel that not having visitors has been a positive thing for my birthing process.	434	1 5	4.15 (1.143)	5	16 (3.7%)	45 (10.4%)	25 (5.8%)	122 (28.1%)	226 (52.1%)
3. I believe that not having visitors has been positive in establishing the mother–newborn bond.	434	1 5	4.37 (0.989)	5	8 (1.8%)	31 (7.1%)	20 (4.6%)	108 (24.9%)	267 (61.5%)
4. I have been lonely during my hospital stay, missing my loved ones. *	434	1 5	2.76 (1.387)		94 (21.7%)	135 (31.1%)	48 (11.1%)	94 (21.7%)	63 (14.5%)
5. I feel that the postpartum period has been more relaxed than in previous deliveries due to the limitations of the visits. **	206	1 5	4.18 (1.057)		9 (4.4%)	8 (3.9%)	21 (10.2%)	66 (32%)	102 (49.5%)
Dimension 2. PCR testing	430	1 5	4.15 (0.824)	4.33					
6. I consider that the performance of a PCR test on admission to hospital is a wise measure due to the pandemic situation in which we find ourselves.	434	1 5	4.16 (1.010)		15 (3.5%)	20 (4.6%)	42 (9.7%)	161 (37.1%)	196 (45.2%)
7. I think that the PCR should have been done also to my companion.	434	1 5	4.19 (1.050)	5	16 (3.7%)	20 (4.6%)	49 (11.3%)	129 (29.7%)	220 (50.7%)
8. Knowing that my PCR was negative has given me peace of mind.	430	1 5	4.09 (1.033)		17 (4%)	12 (2.8%)	73 (17%)	141 (32.8%)	187 (43.5%)
Dimension 3. Mask use.	433	2.25 5	3.98 (0.496)						
9. I consider that the use of the mask should be compulsory only in the case of unknown or positive PCR.	434	1 5	4.07 (1.254)	5	28 (6.5%)	44 (10.1%)	26 (6%)	107 (24.7%)	229 (52.8%)
10. I consider that the use of a mask is a wise measure due to the pandemic situation in which we find ourselves.	434	1 5	3.46 (1.286)		43 (9.9%)	75 (17.3%)	55 (12.7%)	161 (37.1%)	100 (23%)
11. I consider that the use of a mask in the room when they are shared is necessary.	434	1 5	3.68 (1.223)		25 (5.8%)	74 (17.1%)	43 (9.9%)	163 (37.8%)	128 (29.5%)
12. I believe that the use of the mask should be made more flexible at the time of delivery, allowing the woman to remove it.	433	1 5	4.70 (0.697)	5	4 (0.9%)	8 (1.8%)	11 (2.5%)	70 (16.2%)	340 (78.5%)

N—number of participants; Min—minimum; Max—maximum; M—mean; SD—standard deviation; Med—median; 1—strongly disagree; 2—disagree; 3—indifferent; 4—agree; 5—strongly agree; * additional question; and ** additional question only for women with more children.

**Table 3:** Inferential statistics. T-student for having passed COVID-19.

ITEMS	N	Min. Max.	M (DS)	t	gl	*p*		N	M	DS
Dimension 1. Visit restrictions.	434	1 5	4.27 (0.910)	−1.894	432	0.059	YES NO	176 258	4.17 4.34	1.034 0.811
1. I believe that the measure to limit visits is necessary because of the COVID-19 pandemic.	434	1 5	4.29 (0.994)	−3.0678	432	0.002	YES NO	176 258	4.12 4.41	1.122 0.879
2. I feel that not having visitors has been a positive thing for my birthing process.	434	1 5	4.15 (1.143)	−0.7307	432	0.465	YES NO	176 258	4.10 4.18	1.227 1.084
3. I believe that not having visitors has been positive in establishing the mother–newborn bond.	434	1 5	4.37 (0.989)	−1.3153	432	0.189	YES NO	176 258	4.30 4.42	1.087 0.915
4. I have been lonely during my hospital stay, missing my loved ones. *	434	1 5	2.76 (1.387)	−0.0867	432	0.931	YES NO	176 258	2.76 2.77	1.399 1.381
5. I feel that the postpartum period has been more relaxed than in previous deliveries due to the limitations of the visits. **	206	1 5	4.18 (1.057)	−0.0663	204	0.947	YES NO	84 122	4.18 4.19	1.043 1.071
Dimension 2. PCR testing	430	1 5	4.15 (0.824)	−3.146	428	0.002	YES NO	172 258	3.99 4.25	0.886 0.765
6. I consider that the performance of a PCR test on admission to hospital is a wise measure due to the pandemic situation in which we find ourselves.	434	1 5	4.16 (1.010)	−2.9262	432	0.004	YES NO	176 258	3.99 4.28	1.074 0.949
7. I think that the PCR should have been done also to my companion.	434	1 5	4.19 (1.050)	−1.9301	432	0.054	YES NO	176 258	4.07 4.27	1.116 0.996
8. Knowing that my PCR was negative has given me peace of mind.	430	1 5	4.09 (1.033)	−2.5519	428	0.011	YES NO	172 258	3.94 4.19	1.104 0.971
Dimension 3. Mask use.	433	2.25 5	3.98 (0.496)	−0.390	431	0.696	YES NO	176 257	3.96 3.98	0.496 0.497
9. I consider that the use of the mask should be compulsory only in the case of unknown or positive PCR.	434	1 5	4.07 (1.254)	1.9894	432	0.047	YES NO	176 258	4.22 3.97	1.180 1.295
10. I consider that the use of a mask is a wise measure due to the pandemic situation in which we find ourselves.	434	1 5	3.46 (1.286)	−1.0727	432	0.284	YES NO	176 258	3.38 3.52	1.241 1.315
11. I consider that the use of a mask in the room when they are shared is necessary.	434	1 5	3.68 (1.223)	−2.4135	432	0.016	YES NO	176 258	3.51 3.80	1.256 1189
12. I believe that the use of the mask should be made more flexible at the time of delivery, allowing the woman to remove it.	433	1 5	4.70 (0.697)	1.3568	431	0.176	YES NO	176 257	4.75 4.66	0.646 0.729

N—number of participants; Min—minimum; Max—maximum; M—mean; SD—standard deviation; * additional question; and ** additional question only for women with more than one child.

**Table 4:** Inferential statistics. ANOVA for type of delivery.

ITEMS	N	Min. Max.	M (DS)	f	gl	*p*		N	M	DS
Dimension 1. Visit restrictions.	434	1 5	4.27 (0.910)	1.6716	184.6	0.191	EU INS CS	247 96 91	4.34 4.19 4.16	0.887 0.917 0.958
1. I believe that the measure to limit visits is necessary because of the COVID-19 pandemic.	434	1 5	4.29 (0.994)	0.8340	186.5	0.436	EU INS CS	247 96 91	4.35 4.22 4.23	0.980 0.943 1.086
2. I feel that not having visitors has been a positive thing for my birthing process.	434	1 5	4.15 (1.143)	2.1926	181.9	0.115	EU INS CS	247 96 91	4.24 4.07 3.96	1.096 1.145 1.246
3. I believe that not having visitors has been positive in establishing the mother–newborn bond.	434	1 5	4.37 (0.989)	0.9618	179.5	0.384	EU INS CS	247 96 91	4.43 4.29 4.30	0.938 1.025 1.080
4. I have been lonely during my hospital stay, missing my loved ones. *	434	1 5	2.76 (1.387)	3.0793	186.7	0.048	EU INS CS	247 96 91	2.66 2.74 3.08	1.361 1.416 1.392
5. I feel that the postpartum period has been more relaxed than in previous deliveries due to the limitations of the visits. **	206	1 5	4.18 (1.057)	0.2097	52.1	0.812	EU INS CS	146 31 29	4.18 4.10 4.28	1.050 1.193 0.960
Dimension 2. PCR testing	430	1 5	4.15 (0.824)	1.9060	183.1	0.152	EU INS CS	246 93 91	4.13 4.05 4.28	0.803 0.935 0.749
6. I consider that the performance of a PCR test on admission to hospital is a wise measure due to the pandemic situation in which we find ourselves.	434	1 5	4.16 (1.010)	0.6454	194.6	0.526	EU INS CS	247 96 91	4.15 4.10 4.25	1.026 1.090 0.877
7. I think that the PCR should have been done also to my companion.	434	1 5	4.19 (1.050)	2.2726	200.5	0.106	EU INS CS	247 96 91	4.17 4.08 4.36	1.084 1.130 0.837
8. Knowing that my PCR was negative has given me peace of mind.		1 5	4.09 (1.033)	1.2255	176.8	0.296	EU INS CS	246 93 91	4.09 3.97 4.22	0.977 1.174 1.020
Dimension 3. Mask use.	433	2.25 5	3.98 (0.496)	0.7555	190.0	0.471	EU INS CS	246 96 91	3.97 4.02 3.94	0.498 0.466 0.524
9. I consider that the use of the mask should be compulsory only in the case of unknown or positive PCR.	434	1 5	4.07 (1.254)	1.4209	188.6	0.244	EU INS CS	247 96 91	4.06 4.23 3.92	1.244 1.138 1.384
10. I consider that the use of a mask is a wise measure due to the pandemic situation in which we find ourselves.	434	1 5	3.46 (1.286)	0.0682	188.4	0.934	EU INS CS	247 96 91	3.44 3.48 3.49	1.283 1.281 1.311
11. I consider that the use of a mask in the room when they are shared is necessary.	434	1 5	3.68 (1.223)	0.0718	187.0	0.931	EU INS CS	247 96 91	3.69 3.65 3.71	1.212 1.214 1.276
12. I believe that the use of the mask should be made more flexible at the time of delivery, allowing the woman to remove it.		1 5	4.70 (0.697)	0.7445	190.6	0.476	EU INS CS	246 96 91	4.71 4.74 4.62	0.697 0.603 0.786

N—number of participants; Min—minimum; Max—maximum; M—mean; SD—standard deviation; EU—Eutocic delivery; INS—Instrumental delivery; CS—Caesarean section; * additional question; and ** Additional question only for women with more children.

## Data Availability

The data presented in this study are available on request from the corresponding authors.

## Supplementary Materials

The following supporting information can be downloaded at: https://www.mdpi.com/article/10.3390/healthcare11020249/s1, Table S1: Survey’s CVI results.

## References

[1] Mercer R.T. (2006). Nursing support of the process of becoming a mother. J. Obstet. Gynecol. Neonatal Nurs..

[2] Mercer R.T. (2004). Becoming a mother versus maternal role attainment. J. Nurs. Scholarsh..

[3] Ramírez-Peláez H., Rodriguez-Gallego I., Beneficios del acompañamiento a la mujer por parte de su pareja durante el embarazo, el parto y el puerperio en relación con el vínculo paternofilial (2014). Revisión bibliográfica. Matronas. Prof..

[4] Gobierno de España (2020). Documento técnico. Manejo de la mujer embarazada y el recién nacido con COVID-19.

[5] Gobierno de España (2020). ESTRATEGIA ESTATAL CONTRA LA SEGUNDA OLA.

[6] Bradfield Z., Hauck Y., Homer C.S.E., Sweet L., Wilson A.N., Szabo R.A., Wynter K., Vasilevski V., Kuliukas L. (2022). Midwives’ experiences of providing maternity care during the COVID-19 pandemic in Australia. Women Birth.

[7] Hazfiarini A., Zahroh R.I., Akter S., Homer C.S.E., Bohren M.A. (2022). Indonesian midwives’ perspectives on changes in the provision of maternity care during the COVID-19 pandemic: A qualitative study. Midwifery.

[8] (2020). Real Decreto 900/2020, de 9 de octubre, por el que se declara el estado de alarma para responder ante situaciones de especial riesgo por transmisión no controlada de infecciones causadas por el SARS-CoV-2.

[9] (2021). Ley 2/2021, de 29 de marzo, de medidas urgentes de prevención, contención y coordinación para hacer frente a la crisis sanitaria ocasionada por el COVID-19.

[10] (2021). Real Decreto-ley 30/2021, de 23 de diciembre, por el que se adoptan medidas urgentes de prevención y contención para hacer frente a la crisis sanitaria ocasionada por la COVID-19.

[11] Consejería de Salud (2018). Gobierno de la Región de Murcia. LACTANCIA MATERNA, desde el principio..

[12] Comité de Lactancia Materna de la Asociación Española de Pediatría (2017). LACTANCIA MATERNA, EL MEJOR INICIO PARA AMBOS.

[13] Bohren M.A., Hofmeyr G.J., Sakala C., Fukuzawa R.K., Cuthbert A. (2017). Continuous support for women during childbirth. Cochrane Database Syst. Rev..

[14] Barrio-Forné N., Gasch-Gallén, Á (2021). Companionship as a method to reduce anxiety in pregnant women hospitalized during their third trimester. Rev. Esc. Enferm. USP.

[15] Bohren M.A., Berger B.O., Munthe-Kaas H., Tunçalp Ö. (2019). Perceptions and experiences of labour companionship: A qualitative evidence synthesis. Cochrane Database Syst. Rev..

[16] Ministerio de Sanidad y Política Social (2010). Guía de Práctica Clínica sobre la Atención al Parto Normal. https://www.sanidad.gob.es/organizacion/sns/planCalidadSNS/pdf/equidad/guiaPracClinPartoCompleta.pdf.

[17] Ministerio de Sanidad y Consumo (2008). Estrategia de Atención al Parto Normal en el Sistema Nacional de Salud. https://www.sanidad.gob.es/organizacion/sns/planCalidadSNS/pdf/InformeFinalEAPN_revision8marzo2015.pdf.

[18] Ministerio de Sanidad Política Social e Igualdad (2011). Plan de Parto y Nacimiento. https://www.sanidad.gob.es/organizacion/sns/planCalidadSNS/pdf/equidad/planPartoNacimiento.pdf.

[19] WHO (2018). Recomendaciones de la OMS Para Los Cuidados Durante el Parto, Para Una Experiencia de Parto Positive.

[20] WHO (2002). Promoting Effective Perinatal Care 2002 Essential Newborn Care and Breastfeeding Training Modules WHO Regional Office for Europe.

[21] Federación de Asociaciones de Matronas de España (2020). Posicionamiento FAME Covid-19. https://www.federacion-matronas.org/2020/03/23/posicionamiento-fame-covid/=21.

[22] Ministerio de Sanidad, Gobierno de España (2020). Información y pautas generales para mujeres embarazadas en situación de confinamiento. https://www.sanidad.gob.es/profesionales/saludPublica/ccayes/alertasActual/nCov/ciudadania.htm.

[23] WHO, OPS (2020). Cuidar a Mujeres Embarazadas y Recién Nacidos con Confirmación o Sospecha de COVID-19.

[24] Arroyo-Menéndez M., Finkel L. (2019). Encuestas por Internet y nuevos procedimientos muestrales. Panor. Social.

[25] Almanasreh E., Moles R., Chen T.F. (2019). Evaluation of methods used for estimating content validity. Res. Social Adm. Pharm..

[26] Lancet T. (2000). A fifth amendment for the declaration of Helsinki. Lancet.

[27] Olza-Fernández I. (2002). LACTANCIA DESPUÉS DE LA CESÁREA. Personal communication.

[28] Zhang F., Cheng J., Yan S., Wu H., Bai T. (2019). Early Feeding Behaviors and Breastfeeding Outcomes After Cesarean Section. Breastfeed. Med..

[29] Altman M.R., Eagen-Torkko M.K., Mohammed S.A., Kantrowitz-Gordon I., Khosa R.M., Gavin A.R. (2021). The impact of COVID-19 visitor policy restrictions on birthing communities of colour. JAN Lead. Glob. Nurs. Res..

[30] Jackson L., de-Pascalis L., Harrold J.A., Fallon V., Silverio S.A. (2022). Postpartum women’s experiences of social and healthcare professional support during the COVID-19 pandemic: A recurrent cross-sectional thematic analysis. Women Birth.

[31] Rice K., Williams S. (2021). Women’s postpartum experiences in Canada during the COVID-19 pandemic: A qualitative study. CMAJ. Open.

[32] Sweet L., Wilson A.N., Bradfield Z., Hauck Y., Kuliukas L., Homer C.S.E., Szabo R.A., Wynter K., Vasilevski V. (2022). Childbearing women’s experiences of the maternity care system in Australia during the first wave of the COVID-19 pandemic. Women Birth.

[33] Karavadra B., Stockl A., Prosser-Snelling E., Simpson P., Morris E. (2020). Women’s perceptions of COVID-19 and their healthcare experiences: A qualitative thematic analysis of a national survey of pregnant women in the United Kingdom. BMC Pregnancy Childbirth.

[34] Instituto de Salud Carlos III, Departamento de Salud del Gobierno Vasco (2017). Guía Para las Madres que Amamantan. www.guiasalud.es.

[35] Martínez-Cardona J.A., Cordova-Salazar J.K. (2021). Implicaciones de la restricción de las visitas familiares por la pandemia por COVID-19 y el crecimiento-desarrollo del prematuro en la Unidad de Cuidados Intensivos. Aten Primaria.

[36] Balaguer M.S., Nieto M.A., Peressutti S.N. (2018). Acercando las Unidades Neonatales Mediante Vínculos Saludables: Visitas Programadas de Hermanos en Neonatología. Actas II Congreso Internacional de Psicología “CIENCIA Y PROFESIÓN”. Córdoba España.

[37] Bautista-Balbás L., Velasco-Guijarro O., Sandino-Gómez R., Rosado-Palacios M., Gil-Conesa M., Cordero-Castrejón V., Gil Rosado R., Moraleda P.L., Palacios F.P., Parras B.P. (2021). Experiencia de un hospital de media estancia en época COVID-19 con protocolos de visitas. Rev. Esp. Salud Publica.

[38] Consejería de Salud (2021). Estrategia de Detección Precoz, Vigilancia y Control de la COVID-19 Adaptación para el Servicio Murciano de Salud Murcia. https://sede.carm.es/verificardocumentos.

[39] Aranaz-Andrés J.M., Gea-Velázquez de Castro M.T., Vicente-Guijarro J., Beltrán-Peribáñez J., García-Haro M., Valencia-Martín J.L., Bischofberger Valdés C., Grupo de Trabajo COVID-19 del Hospital Universitario Ramón y Cajal (2020). Mascarillas como equipo de protección individual durante la pandemia de COVID-19: Cómo, cuándo y cuáles deben utilizarse. J. Healthc. Qual. Res..

[40] (2022). Resolución de 13 de julio de 2020, de la Secretaría General de la Consejería de Presidencia y Hacienda.

[41] Orden SND/422/2020, de 19 de Mayo, Por la que se Regulan las Condiciones Para el uso Obligatorio de Mascarilla Durante la Situación de Crisis Sanitaria Ocasionada por el COVID-1. https://www.boe.es.

[42] Real Decreto 115/2022, de 8 de Febrero, Por el Que se Modifica la Obligatoriedad del Uso de Mascarillas Durante la Situación de Crisis Sanitaria Ocasionada Por el COVID-19. https://www.boe.es.

[43] Real Decreto 286/2022, de 19 de Abril, por el que se Modifica la Obligatoriedad del Uso de Mascarillas Durante la Situación de Crisis Sanitaria Ocasionada por la COVID-19. https://www.boe.es.

[44] Poon L.C., Yang H., Kapur A., Melamed N., Dao B., Divakar H., McIntyre H.D., Kihara A.B., Ayres-De-Campos D., Ferrazzi E.M. (2020). Global interim guidance on coronavirus disease 2019 (COVID-19) during pregnancy and puerperium from FIGO and allied partners: Information for healthcare professionals. Int. J. Gynaecol Obstet..

