# Supplementary material for: Impact of COVID-19 Restrictions in Childbirth and Puerperium: A Cross-Sectional Study
Source: Healthcare (Basel). 2023 Jan 13;11(2):249. doi: 10.3390/healthcare11020249 (PMC9859232; doi:10.3390/healthcare11020249)
Supplement: Supplementary file 1 [file healthcare-11-00249-s001.zip › healthcare-2157318-supplementary.pdf]

Supplementary Materials

Table S1. CVI results from survey

Item	E.1	E. 2	E.3	E.4	E.5	E.6	E.7	E.8	E.9	E.10	Total	Item-CVI
1	4	4	4	3	4	4	3	4	4	4	10	1.00
2	4	4	4	3	4	4	4	4	4	4	10	1.00
3	4	2	4	3	4	4	4	4	4	4	9	0.9
6	4	2	4	2	4	3	4	3	4	4	8	0.8
7	4	3	2	3	4	2	3	4	4	3	8	0.8
8	4	3	4	4	4	4	2	4	4	4	9	0.9
9	4	4	4	3	4	4	4	4	4	4	10	1.00
10	4	4	4	3	4	4	4	4	4	4	10	1.00
11	4	4	2	3	4	2	4	4	4	4	8	0.8
12	4	4	4	2	4	3	2	4	4	4	8	0.8
Total	10	8	8	9	10	8	8	10	10	10	Total-Scale CVI	0.9
Proportion	1.00	0.8	0.9	0.9	1.00	0.8	0.8	1.00	1.00	1.00		

Proportion: Proportion of experts giving a value of at least 3 to the item.
